# Influence of Head Tissue Conductivity Uncertainties on EEG Dipole Reconstruction

**DOI:** 10.3389/fnins.2019.00531

**Published:** 2019-06-04

**Authors:** Johannes Vorwerk, Ümit Aydin, Carsten H. Wolters, Christopher R. Butson

**Affiliations:** ^1^Scientific Computing & Imaging (SCI) Institute, University of Utah, Salt Lake City, UT, United States; ^2^Institute for Biomagnetism and Biosignalanalysis, University of Münster, Münster, Germany; ^3^Institute of Electrical and Biomedical Engineering, UMIT - University for Health Sciences, Medical Informatics and Technology, Hall in Tirol, Austria; ^4^Multimodal Functional Imaging Lab, Department of Physics and PERFORM Centre, Concordia University, Montreal, QC, Canada; ^5^Otto Creutzfeldt Center for Cognitive and Behavioral Neuroscience, University of Münster, Münster, Germany; ^6^Departments of Biomedical Engineering, Neurology, and Psychiatry, University of Utah, Salt Lake City, UT, United States; ^7^Department of Neurosurgery, Clinical Neurosciences Center, University of Utah, Salt Lake City, UT, United States

**Keywords:** EEG source analysis, EEG dipole reconstruction, head modeling, sensitivity analysis, conductivity uncertainty, conductivity estimation, finite element method, generalized polynomial chaos

## Abstract

Reliable EEG source analysis depends on sufficiently detailed and accurate head models. In this study, we investigate how uncertainties inherent to the experimentally determined conductivity values of the different conductive compartments influence the results of EEG source analysis. In a single source scenario, the superficial and focal somatosensory P20/N20 component, we analyze the influence of varying conductivities on dipole reconstructions using a generalized polynomial chaos (gPC) approach. We find that in particular the conductivity uncertainties for skin and skull have a significant influence on the EEG inverse solution, leading to variations in source localization by several centimeters. The conductivity uncertainties for gray and white matter were found to have little influence on the source localization, but a strong influence on the strength and orientation of the reconstructed source, respectively. As the CSF conductivity is most accurately determined of all conductivities in a realistic head model, CSF conductivity uncertainties had a negligible influence on the source reconstruction. This small uncertainty is a further benefit of distinguishing the CSF in realistic volume conductor models.

## 1. Introduction

Electroencephalography (EEG) source analysis is an important tool in a variety of both clinical and scientific applications to identify the active brain areas that evoke a measured signal (Brette and Destexhe, 2012). In practice, EEG source analysis consists of two problems: simulating the EEG signal evoked by activity in a certain brain region (EEG forward problem) and, based on these simulations, reconstructing the active brain areas underlying a measured EEG signal (EEG inverse problem). The accuracy of the EEG inverse solution depends on various factors, for example the signal-to-noise ratio (SNR) of the measurement (Arridge et al., 2006; Bast et al., 2006), the number of EEG sensors (Lantz et al., 2003; Grieve et al., 2004), the applied inverse method (Pascual-Marqui, 2002; Lucka et al., 2012), or the accuracy of the EEG forward solution (Acar and Makeig, 2013; Cho et al., 2015). Of course, the amount of influence of each of these factors on the source analysis result varies significantly. This study is devoted to investigating how variations of the EEG forward solution due to head tissue conductivity uncertainties influence the results of EEG source analysis.

The computation of forward solutions is based on a forward model, i.e., a discrete representation of the geometry and conductive features of the subject's head. Various studies have investigated the influence of simplifications of forward models both directly on solutions to the forward problem and indirectly on solutions to the inverse problem. Examples include modeling a homogenized skull compartment instead of distinguishing skull compacta and spongiosa or a homogenized brain compartment instead of distinguishing cerebrospinal fluid (CSF), gray matter, and white matter. In this context, both simulation studies that focus on investigating the influence of simplifications of the modeling of a single conductive feature on EEG forward and inverse solutions, e.g., of the skull (Dannhauer et al., 2011; Montes-Restrepo et al., 2014) or of white matter anisotropy (Wolters et al., 2006; Hallez et al., 2008; Güllmar et al., 2010), and that compare the influence of several of these modeling steps on forward solutions (Haueisen et al., 1997; Ramon et al., 2004; Vorwerk et al., 2014; Azizollahi et al., 2016), source analysis of dipole sources (Acar and Makeig, 2013), or source connectivity analysis (Cho et al., 2015) have been conducted.

However, most of these studies have not taken into account that the conductivity values chosen for each modeled compartment are uncertain, i.e., the conductivity values were assumed to be exactly known. The uncertainties for the conductivity values are a consequence of inter- and intrasubject variability and depend, for example, on the age or the disease state of the subject (Haueisen et al., 1997). The effects of the variability of the skull conductivity have already been investigated, and studies have shown the strong influence of varying skull conductivities on source analysis results, for example for the localization of single dipole sources in simulation studies (Dannhauer et al., 2011) or the localization of epileptic spikes (Aydin et al., 2014). The influence of conductivity variations of other compartments, such as skin, gray matter, or white matter, has also been previously evaluated in forward modeling (Haueisen et al., 1997; Azizollahi et al., 2016) and sensitivity studies (Gençer and Acar, 2004; Vallaghé and Clerc, 2009), none of which, however, evaluated actual EEG measurements.

In this study, we investigated and compared the influence of conductivity uncertainties of the skin, skull, CSF, gray matter, and white matter on EEG forward simulations and single dipole reconstructions of the somatosensory evoked potential (SEP) P20/N20 component that was measured with surface EEG. The assumption of a single focal dipole source for this component was previously justified (Allison et al., 1991; Hari et al., 1993; Kakigi, 1994; Fuchs et al., 1998), making it an ideal candidate for a sensitivity study. Furthermore, conductivity calibration approaches based on combined EEG and MEG recordings of the SEP P20/N20 component have been proposed (Fuchs et al., 1998; Aydin et al., 2014). Therefore, a better understanding of the sensitivity of the source localization of the SEP P20/N20 component toward the different tissue conductivities may help to further improve these approaches. We used goal function scans (GFS, Knösche, 1997; Fuchs et al., 1998) as the source reconstruction method.

To obtain statistical distributions of the results of both EEG forward simulations and source reconstructions for uncertain conductivity values, we used Monte Carlo methods. Following the approach of Schmidt et al. (2015) and Schmidt et al. (2013), we used a generalized polynomial chaos (gPC) approach to be able to perform several dipole localizations for varying conductivity values in a short amount of time and to obtain statistical distributions of forward simulation and source reconstruction results. Based on these distributions, we analyzed the influence of conductivity variations on EEG forward simulations and source reconstructions.

## 2. Materials and Methods

### 2.1. SEP Data Acquisition and Preprocessing

**Figure 1:**
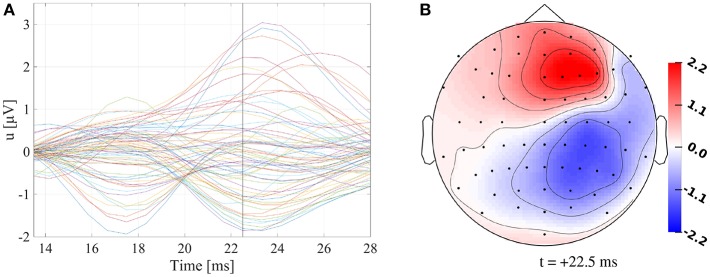
**(A)** Butterfly plot of preprocessed SEP data. Vertical black line marks +22.5 ms. **(B)** Topography plot of preprocessed SEP data at +22.5 ms. Values are indicated in μV.

For a healthy 25-year-old participant, we recorded SEPs evoked by electrical median nerve stimulation of the participant's left wrist using a 74-channel EEG-system (10–10 system) with six additional EOG channels to detect eye movements. The participant signed a written consent form, and all procedures were approved by the ethics committee of the University of Erlangen, Faculty of Medicine on 10.05.2011 (Ref. No. 4453). Prior to the EEG measurement, the electrode positions were digitized using a Polhemus device. Electrical pulses with 0.5 ms duration were used as stimuli, for which the stimulation strength was adjusted until a clear movement of the thumb was visible. The interstimulus interval (ISI) was varied between 350 ms and 450 ms. During one run of 7 min at a frequency of 1,200 Hz, 972 events were recorded and filtered online with a 300 Hz low pass filter. The measurements were filtered using a band pass filter of 20–250 Hz (Buchner et al., 1994) and a notch filter for the line voltage frequency of 50 Hz and its harmonics. The continuous data were cut into epochs of 100 ms before and 200 ms after the stimulus. EEG channels were inspected visually before and after averaging epochs. Channels FC1, F1, C1, and FT7 consistently showed higher variation and amplitudes in comparison to other channels, even on baseline intervals prior to stimuli, and were therefore excluded from the analysis. Epochs with artifacts were excluded using a threshold-based semiautomatic procedure followed by manual inspection. The remaining epochs were averaged, and the P20/N20 component was localized at the peak (i.e., in our experimental setup at +22.5 ms, see Figure 1, Buchner et al., 1994). The resulting signal-to-noise ratio, calculated as the ratio of the power at the peak of the P20/N20 component and the average power in the prestimulus interval from -100 ms to -30 ms, was 7.5.

### 2.2. Head Model Generation

To compute accurate EEG forward solutions using the finite element method (FEM), we constructed a realistic, high-resolution five-compartment (skin, skull, CSF, gray matter, white matter) head model. The compartment interfaces were segmented based on T1- and T2-MRI scans; the T1-weighted image was acquired with an MP-RAGE pulse sequence and the T2-weighted image with an SPC pulse sequence. The MR images were registered and surfaces of the compartment interfaces were generated based on a semiautomatic segmentation of these images. Subsequently, a high-quality tetrahedral mesh was created using TetGen (Si, 2015). The geometry of this model is identical to the high-resolution model *6CA_hr* created in Vorwerk et al. (2014). For a more detailed description of the model generation, we refer the reader to this publication.

Based on the results of Vorwerk et al. (2014) and due to the computational effort of the gPC expansion performed in this study, we introduced two simplifications to the head model. We omitted the distinction between compact and spongy bone, which was shown to have a recognizable effect only for temporal sources (Dannhauer et al., 2011; Vorwerk et al., 2014), and the modeling of white matter anisotropy, which is still afflicted with possible inaccuracies and is furthermore assumed to have only a comparatively small influence for a source as superficial as the somatosensory source investigated here (Acar and Makeig, 2013; Vorwerk et al., 2014). The resulting model contained 2,159,337 vertices.

### 2.3. EEG Source Analysis

Based on this head model, EEG forward solutions were computed using the FEM. More specifically, we applied the St. Venant approach based on comparisons of the accuracy and performance of different FEM approaches for EEG forward computations (Bauer et al., 2015). In the St. Venant approach, a dipole source is approximated by a distribution of electrical monopoles placed on the vertices of the head model neighboring the source position. The charges of the monopoles are determined so that the moments of the distribution of monopoles optimally match those of a dipole at the source position (Buchner et al., 1997). Thereby, even though the distribution of monopoles now extends over the volume of a few mesh elements, the electric field evoked by the distribution of monopoles in sufficient distance from the source is essentially identical to that of the original dipole source. For an exact representation of the dipole moment of the simulated source, the St. Venant approach requires that all monopoles are in a region of the same conductivity (“St. Venant condition”). A placement of monopoles in multiple compartments of different conductivities may affect the numerical accuracy of the EEG forward solutions. For the FEM forward computations, we used SimBio (https://www.mrt.uni-jena.de/simbio/) in combination with an algebraic multigrid conjugate gradient (AMG-CG) solver (Lew et al., 2009).

As the inverse method to reconstruct the measured SEP, we applied goal function scans (also known as single dipole deviation scans, SDDS; Knösche, 1997; Fuchs et al., 1998). For a predefined set of possible source locations, source direction and strength for a single dipole source at each position are fitted to optimally explain the measured data, i.e., to maximize the goodness of fit (GoF).

For a measurement *u*_meas_, the GoF for each source position **x**_*i*_ is computed via

(1) $$\begin{array}{r} GoF\left({\text{x}}_{i}\right)=1-{\left(\frac{\|u_{\text{meas}}-L\left({\text{x}}_{i}\right)L^{+}\left({\text{x}}_{i}\right)u_{\text{meas}}{\|}_{2}}{\|u_{\text{meas}}{\|}_{2}}\right)}^{2}, \end{array}$$

where ‖·‖_2_ is the Euclidian norm, *L*(**x**_*i*_) is the *#sensors* × 3 leadfield matrix for position **x**_*i*_, i.e., a matrix containing the forward simulation results for dipoles with moments oriented in each of the three cartesian directions at the source position, and *L*^+^ its Moore-Penrose inverse. The source position with the overall maximal GoF is adopted as the source location.

If the measured signal is dominated by a single dipolar source, the GFS reconstructs the optimal source location within the spatial accuracy of the source space. The assumption of a single dipole source on which the GFS is based is a strong prior; however, it is justified for the reconstruction of the P20/N20 SEP component (Allison et al., 1991; Hari et al., 1993; Kakigi, 1994; Fuchs et al., 1998).

When applying GFS, the leadfield matrices for all source positions of the chosen source space can be precomputed. Since the EEG forward problem is linear, these simulations are sufficient to calculate forward solutions for dipoles with arbitrary orientation and strength, so that no additional EEG forward computations have to be performed during the dipole reconstruction.

We generated a source space using a two-step approach. First, we performed a GFS with a source space that captured the whole gray matter compartment, where we made sure that the sources were placed at a sufficient distance to the gray matter/CSF as well as the gray/white matter interface to avoid any numerical inaccuracies, and FEM forward solutions computed using the previously generated five-compartment head model with literature values for the conductivities (see Table 1). Afterwards, we generated source positions on a cubic grid centered around this initially reconstructed source position. As we had already obtained an initial guess for the source position, we restricted the size of the grid to 30 mm in each direction. For these dimensions of the source space, no sources were localized on the boundary of the grid in our analysis, showing that the chosen grid dimensions were sufficiently large. Thus, we were able to choose a relatively fine grid resolution of 1.5 mm, resulting in 9,261 source positions. We allowed sources in both the gray and white matter compartments, following the approach taken by Rullmann et al. (2009) and Lanfer et al. (2012), to retain a dense source space that allows variations of the source position in all three spatial dimensions, while preventing large jumps of the source localizations with changing conductivity values due to gaps in the source space. We only removed source positions that were too close to the CSF/brain interface to avoid numerical inaccuracies due to the large conductivity difference, but kept source positions close to the gray/white matter interface. This approach does not respect the St. Venant condition at the gray/white matter boundary, which could affect the results of source localizations. However, the studies of Rullmann et al. (2009) and Lanfer et al. (2012) did not show any effects due to this source space construction, and we also found no indications that such inaccuracies affected the results of our analysis. In the end, 7,483 valid source positions remained.

**Table 1:** Tissue conductivity intervals [mS/m].

**Tissue**	**Min**.	**Max**.	**Standard**	**References**
Skin	280.0	870.0	430.0	Haueisen et al., 1997; Ramon et al., 2004
Skull	1.6	33.0	10.0	Akhtari et al., 2002; Hoekema et al., 2003; Dannhauer et al., 2011
CSF	1769.6	1810.4	1790.0	Baumann et al., 1997
GM	220.0	670.0	330.0	Haueisen et al., 1997; Ramon et al., 2004
WM	90.0	290.0	140.0	Haueisen et al., 1997; Ramon et al., 2004

### 2.4. Tissue Conductivity Distributions

To estimate the influence of tissue conductivity uncertainties on EEG dipole localizations, we had to obtain probability distributions for the conductivity values of the different conductive compartments. We chose a uniform distribution for each conductivity value to account for the lack of further data besides minimal and maximal conductivity (Schmidt et al., 2013, 2015). To define upper and lower bounds for the conductivity of each tissue, we followed the assumptions of Haueisen et al. (1997) and Schmidt et al. (2015) for the conductivities of skin, skull, gray matter, and white matter, assuming upper and lower bounds of approximately ±50% of the mean resistivity. For the skull conductivity, we relied on the literature research performed by Aydin et al. (2014), who found a minimal value of 1.6 mS/m (Akhtari et al., 2002) and a maximal value of 33.0 mS/m (Hoekema et al., 2003). The upper and lower bounds for the CSF conductivity were based on the measurements of Baumann et al. (1997), who found a CSF conductivity of 1790 mS/m at body temperature with a maximal standard deviation of 21 mS/m for frequencies below 500 Hz. We used the 99% confidence interval, which is 1790 ± 20.4 mS/m. Therefore, the relative size of the range in which the CSF conductivity could vary was much smaller than for the other conductivities. Table 1 gives an overview of the conductivity intervals.

### 2.5. Generalized Polynomial Chaos Expansions

This study aimed to obtain and analyze (probability) distributions of the results of both EEG forward simulations and EEG source analysis for uncertain conductivity values. Due to the nontrivial relation between EEG forward solutions and changes of the conductivity values, such probability distributions cannot be obtained analytically but can be only approximated, for example, using Monte Carlo methods. In our experiments, several thousand conductivity values for either a single compartment or for four compartments (skin, skull, gray matter, white matter) at the same time were randomly drawn based on the assumed probability distribution for each conductivity (see Table 1) to subsequently obtain statistical probability distributions for the outcome parameters, e.g., electrode potentials or source localizations.

For each change of the conductivity values, new EEG forward solutions have to be computed. Thus, for source analysis using GFSs, a new leadfield matrix for each randomly drawn conductivity value has to be obtained. Given the sample size used in section 2.6, 10,000 leadfield matrices, each requiring 7,483 × 3 forward solutions, would have to be computed to estimate the influence of the variations of a single conductivity. As the (unparallelized) computation of each leadfield matrix takes about 40 min and requires the allocation of 10 GB of RAM for the used combination of solver method and head model, the overall computation time for 50,000 leadfield matrices (10,000 for each of the uni- and multivariate distributions) would be about 1,400 days. Even though this time could be reduced through parallel computations, these computations remain very (or too) costly. Instead, we applied gPC expansions, which have previously been successfully applied for bioelectric field computations (Schmidt et al., 2013, 2015). Based on exact computations of leadfield matrices for specific conductivity values, these expansions allow us to rapidly compute an interpolated EEG leadfield matrix for a randomly drawn set of conductivities, thereby clearly reducing the number of leadfields that has to be computed with the FEM. In our case, instead of computing 50,000 leadfield matrices, fewer than 500 leadfield matrices had to be computed. The additional time effort for setting up and evaluating the gPC expansions was small in comparison to the time effort for the computation of the leadfield matrices.

We used Legendre polynomials as basis polynomials for the gPC, which are the optimal choice for uniformly distributed random variables as assumed for the conductivities in our study (Schmidt et al., 2013), and Smolyak sparse grids for the numerical integration (Gerstner and Griebel, 1998). The freely available toolbox *UQLab* (Marelli and Sudret, 2014) was used for all computations involving the gPC expansions. We chose a maximal polynomial degree of four in our computations, which can be assumed to be sufficient considering the recommendations of Schmidt et al. (2013, 2015) and Sudret et al. (2006). To evaluate the accuracy of the gPC expansions, we computed RDM and lnMAG errors (Meijs et al., 1989; Güllmar et al., 2010) for leadfields generated using the gPC expansion in comparison to exact leadfield computations with the St. Venant FEM. The RDM measures the topography error (0 ≤ RDM ≤ 2, 0 corresponds to no error and 2 to maximal error) and the lnMAG the magnitude error (−∞ ≤ lnMAG ≤ ∞, 0 corresponds to no error and ±∞ to maximal error). We randomly drew 10 sets of conductivity values for the multivariate distribution, i.e., skin, skull, gray matter, and white matter conductivities were considered uncertain (see section 2.6). Over all sets of conductivity values and all source positions (see section 2.3), we found a maximal RDM of 0.0165 (mean 0.0022) and a maximal absolute value of the lnMAG of 0.0157 (mean 0.0024). These errors are within or below the range of the numerical accuracy of FEM forward solutions in realistic head models (Vorwerk et al., 2014).

### 2.6. Uncertainty Quantification Experiments

We computed univariate gPC expansions as described in section 2.3, i.e., only one conductivity parameter was varied according to its probability distribution, whereas the conductivities for the other compartments were kept at their commonly used literature value (see Table 1). For each conductivity value, 10,000 random samples were drawn based on the distribution described in section 2.4, and the corresponding 10,000 leadfield matrices were calculated using the respective gPC expansions. Based on these leadfield matrices, we estimated probability distributions for the EEG forward solutions. Performing source analysis based on each of these leadfield matrices, we also estimated the distribution of single dipole reconstruction results (see section 2.7).

To reduce the computational effort and due to the negligible influence on the results of both forward and inverse computations for the selected conductivity range, we consecutively dropped the CSF conductivity as a source of possible uncertainty and continued with only the four conductivities of skin, skull, gray matter, and white matter considered as uncertain. We computed a multivariate gPC expansion for these four conductivities, i.e., all four conductivities were considered uncertain at the same time. Based on this gPC expansion, we generated 10,000 leadfield matrices for randomly drawn sets of conductivity values.

### 2.7. Data Evaluation and Visualization

Based on the leadfield matrices that we obtained from the gPC expansions, we evaluated the effects of tissue conductivity uncertainties on EEG source analysis. First, we briefly analyzed the influence of conductivity changes on the EEG forward problem for a dipole representing the P20/N20 SEP component. Subsequently, we analyzed in more depth how these changes of the forward solution affect the results of EEG source analysis for this SEP component.

#### 2.7.1. Influence of Tissue Conductivity Uncertainties on EEG Forward Solutions

**Figure 2:**
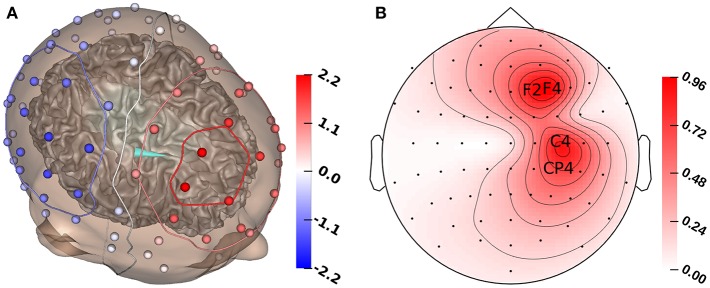
**(A)** GFS source reconstruction (turquoise cone) and simulated electrode voltages for reconstructed source. **(B)** Standard deviation for each electrode for the multivariate distribution. The electrodes with the highest standard deviation (F2, F4, C4, CP4) are marked with their labels. All values in μV.

To analyze the influence of tissue conductivity uncertainties on EEG forward solutions, we evaluated the change of the simulated electrode potentials with varying conductivity values for a single, fixed source. As source position, we chose the result of our initial source localization with literature conductivity values (see section 2.3, Figure 2A), so that the source position is by construction located inside the gray matter compartment with a sufficient distance to the CSF and white matter compartment. To quantify the influence of changes in conductivity of each compartment considered in the multivariate distribution on the EEG forward solution, we computed the first- and second-order Sobol indices for the simulated electrode potentials for this fixed dipole source (Sobol, 2001).

The Sobol indices measure the amount of the overall variance 𝕍 of an outcome variable that can be explained by the conditional variance 𝕍(*i*, …, *j*) caused by variations of only one or more (for higher orders) parameters *i*, …, *j*. In other words, the conditional variance measures the variance of the outcome variable that results exclusively from the simultaneous variation of the parameters *i*, …, *j*, whereas the effects of variations of subsets of these parameters are subtracted in the computation of 𝕍(*i*, …, *j*). The Sobol index *S*(*i*, …, *j*) is thus defined by

(2) $$\begin{array}{r} S\left(i,\ldots ,j\right)=\frac{𝕍\left(i,\ldots ,j\right)}{𝕍}. \end{array}$$

These indices can be used as a measure of the influence of changes of a certain conductivity on the simulation result. The sum of all Sobol indices is 1 (or 100% if indicated in %). In our analysis, we calculate the Sobol indices with the electrode voltages as outcome variables and the tissue conductivities as parameters to determine the influence of changes of each conductivity on the electrode voltages.

The Sobol indices can be computed through Monte Carlo simulations (Sobol, 2001). However, this approach is computationally challenging, requiring a large number of evaluations (Xiu, 2010). Instead, we exploit that the Sobol indices can be directly derived from the coefficients of the gPC expansion (Sudret, 2008). In our study, we made use of the coefficient-based approach already implemented in UQLab.

#### 2.7.2. Influence of Tissue Conductivity Uncertainties on EEG Dipole Reconstruction

We generated multiple graphs to analyze how different parameters of the localized source, such as the GoF, source depth, orientation, or strength, depend on the varying conductivities. The source depth, i.e., the distance of the source location to the inner skull surface, is of great interest for our evaluations, because previous studies showed that the depth of single-source localizations is clearly affected by changes of the (skull) conductivity. For example, see Aydin et al. (2014) for source reconstructions of epileptic spikes in the temporal lobe or Pohlmeier et al. (1997) in a simulation study for sources distributed on the cortex surface.

Together with the analysis of changes in source strength, orientation, and depth, we display the GoF, which is often used in source analysis scenarios to measure how well a reconstructed source explains the measurement data. By construction of *L*^+^, the result of the GFS minimizes the *L*_2_-norm $\|u_{\text{meas}}-LL^{+}u_{\text{meas}}{\|}_{2}$ and thereby maximizes the GoF for the given set of sources. Changes of the GoF for varying conductivities demonstrate how far these conductivity changes lead to changes of the EEG signal that cannot be compensated for by changes of source strength, orientation, or location (depending on whether a fixed, rotating, or moving dipole is reconstructed).

To measure the changes of source orientation, we computed a coordinate system with the direction of the orientation of the original source reconstruction (see Figure 2A) as the first basis vector, a vector with a radial orientation pointing from the source position toward the inner skull surface as the second basis vector, and a vector that is pointing toward the interhemispheric fissure as the third basis vector. These vectors were orthonormalized using the Gram-Schmidt process. In our results, the azimuthal angle φ indicates a tangential variation of the dipole direction (φ > 0 corresponds to a direction change toward the interhemispheric fissure), and an increase of the elevation angle ϑ indicates a shift of the source toward a more radial direction.

As we did not have an a priori assumption regarding the correlation between source reconstruction results and the randomly drawn conductivity values, we used scatter plots showing these parameters as a function of the tissue conductivity to visualize the results. To show the results of all univariate distributions for one parameter in one graph, we rescaled the conductivities to the interval from 0 to 1. To visualize the simultaneous influence of multiple conductivities on the results of the source localization, we additionally created an image plot with contours.

## 3. Results

### 3.1. Influence of Head Tissue Conductivity Uncertainties on EEG Forward Solutions

Figure 1A shows that the P20/N20 component of the measured SEP has a dipolar pattern, justifying the reconstruction using a single dipolar source as done in our study using GFSs. The result of a single dipole reconstruction using a GFS for standard literature conductivity values (see section 2.3, Table 1) is visualized in Figure 2A, by construction the source is localized in the gray matter. As the first step, we analyze the influence of tissue conductivity uncertainties on the EEG forward solution for this source reconstruction. Figure 2B shows the standard deviation of each electrode voltage for the multivariate distribution, which helps to identify the electrode positions most affected by tissue conductivity uncertainties. We find the highest variances for the electrodes with the largest absolute values, such as F2, F4, C4, and CP4. Electrodes with small absolute values, e.g., those in the deep right temporal or the left occipital region, also show a low variance.

**Figure 3:**
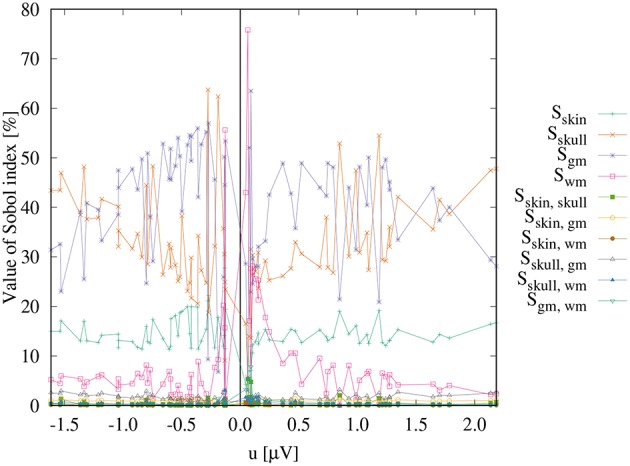
Sobol indices as a function of the electrode voltages.

Figure 3 shows the first- and second-order Sobol indices (see section 2.7.1) for all electrodes with the electrodes sorted according to voltage. The Sobol indices for the two most positive (*F4, F2*) and the two most negative electrodes (*C4, CP4*) are additionally listed in Table 2 (see Figure 2B for the locations of these electrodes). Table 2 shows that the voltage changes for the four electrodes we considered are clearly dominated by the uncertainty of the skull conductivity, as the Sobol index *S*_skull_ is above 40% for all four electrodes, which means that 40% of the voltage variation for these electrodes is caused by skull conductivity uncertainties. We further find a notable influence of gray matter and skin conductivity uncertainties, with Sobol indices *S*_gm_ between 23% and 31% and *S*_skin_ in the range from 15% to 17%. The Sobol indices for the white matter conductivity, *S*_wm_, are at about 2% for the two most positive electrodes, F4 and F2, which are located anterior to the source, but above 5% for the two most negative electrodes C4 and CP4, which are located posterior to the source. This effect can possibly be explained by the position of the dipole source on the anterior side of the postcentral gyrus, so that volume conduction through the white matter (of the gyrus) has a stronger effect on the posterior located electrodes. For the second-order indices, we find a notable influence of the interaction between skull and gray matter conductivity, *S*_skull, gm_, and the interaction between skin and gray matter conductivity, *S*_skin, gm_, which are for all four electrodes larger than 2% and 1%, respectively. In sum, for all four electrodes, the first- and second-order Sobol indices explain at least 99.8% of the variance of the electrode voltages.

**Table 2:** First- and second-order Sobol indices.

**Parameter**	**F4 (%)**	**F2 (%)**	**C4 (%)**	**CP4 (%)**
*S*_skin_	16.7	16.4	17.1	15.0
*S*_skull_	47.8	47.5	46.9	43.4
*S*_gm_	28.1	29.4	23.0	31.4
*S*_wm_	2.3	2.2	6.0	5.2
*S*_skin, skull_	0.7	0.5	1.4	0.4
*S*_skin, gm_	1.1	1.0	1.2	1.1
*S*_skin, wm_	0.1	0.1	0.3	0.1
*S*_skull, gm_	2.7	2.5	2.9	2.6
*S*_skull, wm_	0.3	0.3	0.8	0.5
*S*_gm, wm_	0.1	0.2	0.3	0.3

Figure 3 shows that the distribution of the Sobol indices changes with decreasing absolute values of the electrode voltages. The share of *S*_skull_ decreases, whereas especially that of *S*_gm_ increases. For the electrodes with potentials in the range from -0.2 μV to 0.2 μV, also some high values of *S*_wm_ are observed.

Whereas it can be assumed that the changes of the electrode voltages that are evaluated with the Sobol indices influence the source reconstruction, it is possible that some of these changes only affect the magnitude of the signal but not the topography and would thereby have no influence on the reconstructed source location or orientation. This effect is particularly conceivable for the gray matter conductivity, as the source is located in the gray matter compartment. In this case, the sole interpretation of the Sobol indices could lead to an over- or underestimation of the effects of a certain conductivity on the EEG dipole reconstruction. Thus, it is of interest to evaluate how (much) the observed variations affect the inverse solutions in our further analysis.

### 3.2. Influence of Head Tissue Conductivity Uncertainties on EEG Dipole Reconstruction

**Figure 4:**
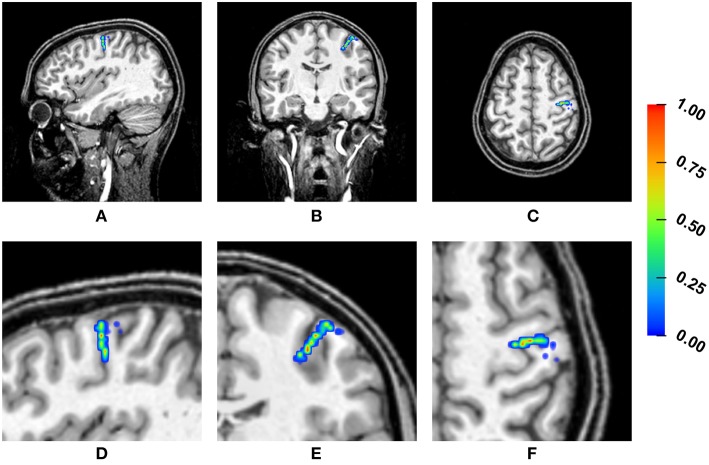
Visualization of the source localizations for the multivariate distribution overlaid on the T1-MRI showing the full head **(A–C)** and a detail around the source locations **(D–F)**. From left to right showing sagittal **(A,D)**, coronal **(B,E)**, and axial slices **(C,F)**. Color bar ranges from low frequency of source localizations (blue) to high frequency of source localizations (red). Values are normalized to the maximum for each slice.

Figure 4 shows a heat map visualization of the reconstructed source locations for the multivariate distribution. For all conductivity combinations, the sources are localized in the somatosensory cortex, as far as can be ascertained (best visible in Figures 4B,E due to the small extent of the distribution of source localizations in anterior-posterior direction), and, except for a few outliers, the sources are localized in the gray matter compartment. However, the location of the sources varies notably. The main variation of the source locations occurs in a direction about orthogonal to the inner surface of the skull, i.e., the depth of the source changes, with only a few outliers for very superficial source positions. Little variation is observed in the tangential directions, so that the distribution of the source locations is stretched out on a virtual radius from the center of the brain toward the inner skull surface, but it is focal in directions tangential to this radius. Therefore, the influence of the conductivity uncertainties on the depth of the source, i.e., the distance to the inner skull surface, is one of the main foci in the following statistical evaluations.

#### 3.2.1. Influence of Single Conductivity Uncertainties

The results of the univariate gPC expansions allow us to analyze how the uncertainty of each conductivity affects the result of the EEG dipole localization. By performing both a fixed (fixed position, fixed orientation, and free strength), a rotating (fixed position, free orientation, and free strength), and a moving GFS (free position, free orientation, and free strength), we successively increase the number of degrees of freedom in our model. To assess the influence of the conductivity uncertainties of each tissue on the source reconstruction, we display the change of the degree of freedom that is added for each step (source strength, elevation angle, source depth) and the GoF side-by-side.

**Figure 5:**
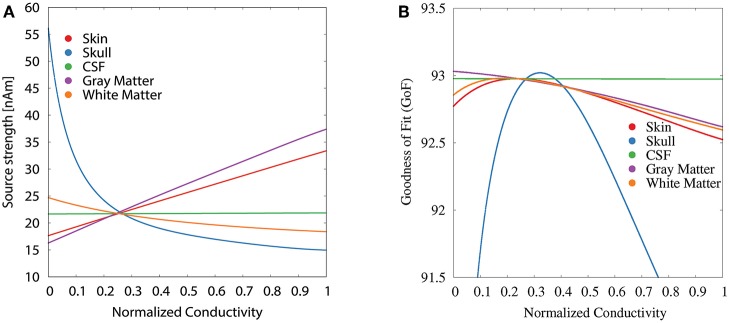
Scatter plots of source strength **(A)** and GoF **(B)** for GFS with fixed dipole location and orientation as a function of the tissue conductivity for univariate distributions. Dipole location and orientation are chosen according to the initial dipole reconstruction (see section 2.3). Conductivities are normalized to the interval from 0 to 1 for clarity of the visualization.

For the GFS with fixed dipole location and orientation (Figure 5), we see the strongest changes in source strength for variations of skull, skin, and gray matter conductivity, whereas we additionally see strong changes in GoF for variations of the white matter conductivity; changes of the CSF conductivity show no influence at all for both measures. For skin and gray matter conductivities, we find an approximately linear relation between conductivity and source strength, whereas we find the inverse behavior for skull and white matter conductivities.

**Figure 6:**
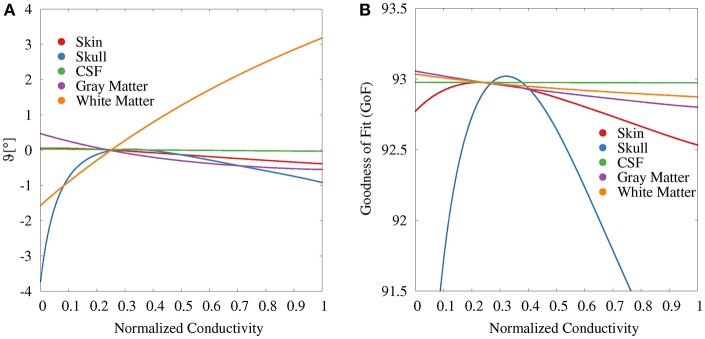
Scatter plots of change in elevation angle (ϑ, **A**) and GoF **(B)** for GFS with fixed dipole location and free orientation as a function of the tissue conductivity for univariate distributions. Dipole location is chosen according to the initial dipole reconstruction (see section 2.3). Conductivities are normalized to the interval from 0 to 1 for clarity of the visualization.

For the GFS with fixed position and free orientation (Figure 6A), we find the strongest influence for white matter and skull conductivities. With regard to the source orientation, we only depicted the results for the elevation angle, i.e., a change toward a more or less radial source orientation, since these showed the strongest effects. A higher white matter conductivity results in a more radial reconstructed source orientation. Summing up the change in elevation and azimuth angle, white matter conductivity uncertainties cause an overall orientation change of about 5°. Changes in the gray matter conductivity have the opposite (but clearly weaker) effect. Gray matter conductivity uncertainties cause an overall orientation change of about 2°. Very low values of the skull conductivity lead to a slightly more tangential and outward-facing source orientation (see Supplementary Figure S1), resulting in an overall orientation change of maximally 3°. The skin conductivity has a negligible influence on the source orientation; the CSF conductivity has no observable influence at all.

The variations of the GoF for white and gray matter conductivities are reduced for the GFS with free orientation (Figure 6B), whereas the values for skull and skin conductivity remain nearly unchanged with respect to the GFS with fixed orientation. This shows that changes of skull and skin conductivity result in changes of the topography of the EEG forward solution that cannot be compensated for by changes of source orientation and strength.

**Figure 7:**
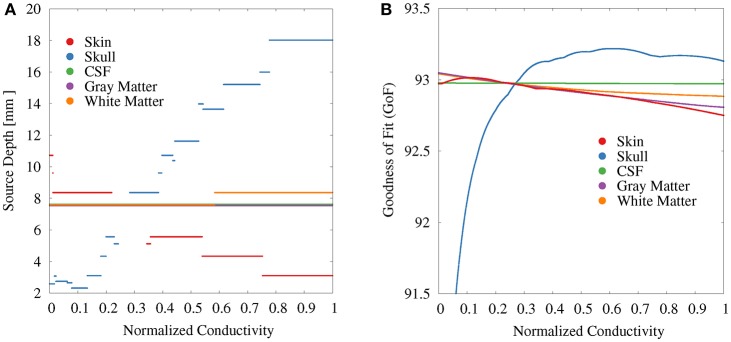
Scatter plots of source depth **(A)** and GoF **(B)** for GFS with free location and orientation as a function of the tissue conductivity for univariate distributions. Conductivities are normalized to the interval from 0 to 1 for clarity of the visualization. Plots for CSF and gray matter are slightly shifted in positive and negative y-direction, respectively, since sources for CSF, gray matter, and partially white matter are localized at the same depth.

For the GFS with free position and orientation, we again find the strongest variations with regard to the source depth for the skull conductivity (Figure 7). These values vary between 18 mm at high conductivities to slightly more than 2 mm at the lowest conductivities (Figure 7A). At a depth of 2 mm, a floor effect occurs, which is the result of excluding source positions that lie outside of the gray and white matter compartments. Instead, a further reduction of the skull conductivity results in more posterior source localizations. For the skin conductivity, the relation between conductivity and source depth is inverted. We find a source depth of about 11 mm for the lowest conductivities, which decreases to about 3 mm with increasing skin conductivity. The source position—and in consequence also the source depth—remains completely unchanged at about 8 mm when the gray matter and CSF conductivities are varied and changes by less than 1 mm when the white matter conductivity is varied.

The variation of the GoF for skull and skin conductivities is clearly reduced for the GFS with free position and orientation. There remains a decrease of the GoF for low skull conductivities, which has the same reason as the floor effect for the source depth, i.e., sources already being localized as superficial as possible at the brain/CSF boundary. For CSF, gray matter, and white matter conductivities the relation between changing conductivities and the GoF is nearly unchanged compared to the GFS with fixed position and free orientation.

These observations suggest that the variations of the EEG forward solution topography caused by uncertainties of skin (less) and skull (more) conductivities mainly result in changes of the source localization whereas the variations of the EEG forward solution topography caused by uncertainties of the gray and white matter conductivities lead to a change in the radial orientation component of the reconstructed source.

#### 3.2.2. Influence of Conductivity Uncertainties on Multivariate Distribution

**Figure 8:**
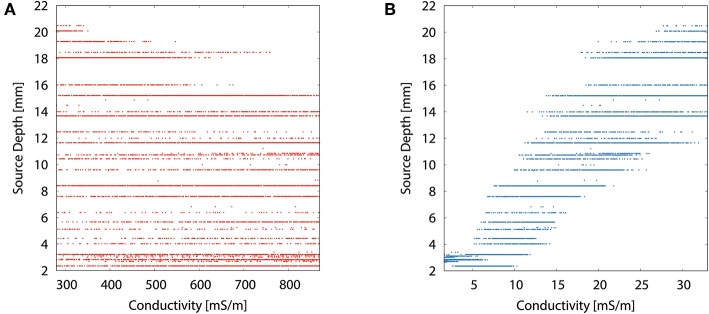
Scatter plots of source depth as a function of the tissue conductivity for the multivariate distribution for skin **(A)** and skull **(B)**.

The scatter plots of source depth as a function of the different tissue conductivities for the multivariate distribution underline the dominance of the skull conductivity with regard to the reconstructed source location, especially to its depth. Figure 8B resembles the curve of the univariate distribution for the skull conductivity (see Figure 7A). However, due to the uncertainty of the other tissue conductivities, a variation of about 10 mm in reconstructed source depth remains for a fixed skull conductivity. In Figure 8A, it can be seen that the deepest source positions are effectively ruled out for high skin conductivities, but due to the uncertainty of the other tissue conductivities the source depth still varies by about 13 mm for the highest skin conductivity. For the other tissue conductivities, only minor or no trends are observable (results not depicted).

Performing a similar analysis for changes of the source orientation does not allow any conclusions. The changes in source orientation caused by variations of gray or white matter conductivities are not strong enough to be observable in the scatter plots, since they are overlaid by changes of the source orientation caused by variations of the source position due to changing skin and skull conductivities (results not depicted).

**Figure 9:**
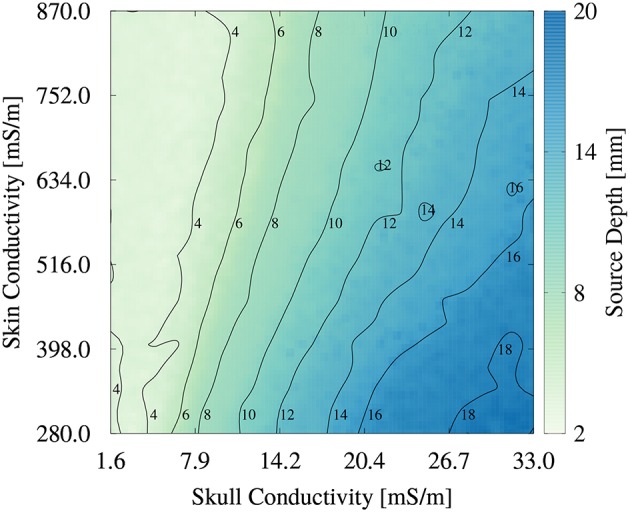
Image plot of source depth as a function of skin and skull conductivities for the multivariate distribution. Contours mark isolines of the source depth.

To better understand the higher order relationship between skin and skull conductivity and source depth, we generated an image plot of the source depth as a function of skull and skin conductivities in Figure 9. For low skull conductivities of up to about 10 mS/m, the influence of the skin conductivity is low, underlining the findings from Figure 8. The contours are nearly vertically oriented and almost parallel to each other. The small tilt of the contours at these conductivities corresponds to the small spread of the scatter plot in Figure 8B at low skull conductivities and source depths. However, the tilted contours also show that small increases of the skull conductivity can be compensated for by an increase of the skin conductivity to result in an unchanged source depth. For higher skull conductivities, the tilt of the contours increases, which corresponds to a higher influence of the skin conductivity on the source depth. This increasing tilt results in an almost diagonal contour for a source depth of 16 mm, which indicates an equal influence of skull and skin conductivity. This result is in line with the interpretation of Figure 8A that the value of the skin conductivity influences the maximal source depth. The smooth contours found in Figure 9 confirm the negligible influence of the remaining tissue conductivities on the source depth.

**Figure 10:**
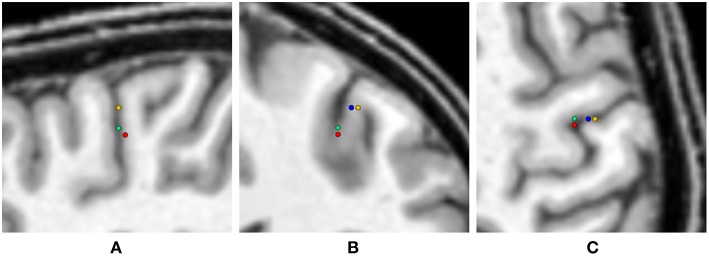
Visualization of the source locations with maximal GoF for the uni- and multivariate distributions overlaid on the T1-MRI (blue - skin, green - skull, yellow - CSF, gray matter, white matter, red - multivariate). From left to right showing sagittal **(A)**, coronal **(B)**, and axial slice **(C)**. In the sagittal slice **(A)** the visualizations for skin and CSF, gray matter, and white matter are overlaying.

To summarize the results of this study, Table 3 lists the maximal GoF and the corresponding conductivity values for the GFS with free position and orientation for each uni- and multivariate distribution, and Figure 10 shows the corresponding source localizations. It has to be noted that none of the values found in Table 3 should be understood as an optimal value to be used in future studies—they just reflect the optimal conductivity values for this specific head and source model to explain the evaluated SEP component, including any kind of measurement noise.

**Table 3:** Optimal GoF achieved for uni- and multivariate distributions and corresponding conductivity values [mS/m].

**Distribution**	**GoF**	**SD(GoF)**	**σ_skin_**	**σ_skull_**	**σ_csf_**	**σ_gm_**	**σ_wm_**
uni skin	93.01	0.08	**346.8**	10.0	1790.0	330.0	140.0
uni skull	93.22	0.74	430.0	**20.4**	1790.0	330.0	140.0
uni csf	92.98	0.00	430.0	10.0	**1769.6**	330.0	140.0
uni gm	93.05	0.07	430.0	10.0	1790.0	**220.0**	140.0
uni wm	93.04	0.04	430.0	10.0	1790.0	330.0	**90.0**
Multi	93.67	0.88	**811.6**	**31.2**	1790.0	**381.7**	**90.8**

*Bold face marks the conductivities that were considered uncertain*.

As expected, the highest overall GoF of 93.67 is reached for the multivariate distribution. The corresponding source position is the deepest of all four source locations evaluated in Figure 10. This GoF is found for a high skin and skull conductivity, a gray matter conductivity close to the literature value, and a low white matter conductivity. Taking into account the results shown in Figure 9, it can be assumed that the high skin and skull conductivity partially compensate each other with regard to the source depth, whereas the change of the white matter conductivity compensates for the orientation of the reconstructed source.

The next highest GoF is found for the skull conductivity at a conductivity of 20 mS/m, which is about twice the literature value. The source is found close to the source location for the multivariate distribution, but not quite as deep. The source locations with the highest GoF for skin, CSF, gray matter, and white matter are all found at similar positions that are clearly more superficial than the position found for the multivariate distribution. Among these four conductivities, the highest GoF is found for a gray matter conductivity at the lower bound of the interval (220 mS/m); a nearly identical GoF is found for a white matter conductivity at the lower bound of the interval (90 mS/m). The slightly higher GoFs for gray matter and white matter conductivities compared to skin and CSF conductivities could possibly be a result of the influence of gray and white matter conductivity on the source orientation. However, the difference in optimal GoF among these four conductivities is less than 0.1 and thereby not meaningful.

Whereas these minimal changes of GoF are not remarkable in practice, the corresponding source locations differ significantly, since the distance between the deepest source position, which is found for the multivariate distribution, and the most superficial source position, found for the univariate distributions of gray matter, white matter, and CSF conductivities, is more than 10 mm.

## 4. Discussion

In this study, we have evaluated the impact of conductivity uncertainties in a five-compartment head model on the solution of the EEG forward problem and on EEG single dipole reconstructions on the example of the SEP P20/N20 component. The main findings are:

- Tissue conductivity uncertainties may lead to significant changes in both reconstructed source location and orientation for single dipole localizations of the SEP P20/N20 component (Figures 4, 10). These changes are not necessarily reflected in measures that are used to evaluate the quality of a source reconstruction, such as the GoF (i.e., the *L*_2_-error).
- Skull and gray matter conductivity uncertainties have the strongest influence on the EEG forward solution. The Sobol indices of the four electrodes with the most variability suggest that these uncertainties cause up to 75% of the overall variation of the EEG forward solution for the electrodes with the highest absolute values in voltage (Figure 3, Table 2).
- Skull and skin conductivity uncertainties have the strongest influence on the single dipole localization. Skull conductivity uncertainties may result in changes in source localization of up to 2 cm; skin conductivity uncertainties in changes of up to 1 cm (Figure 7A). The strong influence of the gray matter conductivity on the EEG forward solution is reflected only in the strength of the reconstructed source (Figure 5A), but not the location.
- Due to having opposite effects on the source depth (Figure 7A), simultaneous changes of skin and skull conductivities may partially eliminate each other when performing single dipole reconstructions (Figure 9, Table 3).
- Gray and white matter conductivity uncertainties barely affect the reconstructed dipole location, but white matter conductivity uncertainties lead to variations in the reconstructed dipole orientation of more than 5° (Figure 6A).
- The Sobol indices for the four electrodes with the highest variability (Table 2) predict the strong influence of skull conductivity uncertainties on the inverse solution. However, the strong influence of gray matter conductivity uncertainties on the EEG forward solution, with Sobol indices of up to 31%, affects only the reconstructed source strength, whereas skin conductivity uncertainties have a much stronger influence on the source localization, despite Sobol indices below 17%. These results show that the isolated interpretation of the Sobol indices is not sufficient to determine the influence of the uncertainties of the different tissue conductivities on the EEG source reconstruction. A separate computation of Sobol indices for changes of signal topography and magnitude could possibly overcome this deficit.
- The small CSF conductivity uncertainties do not affect the result of the dipole reconstruction. However, not modeling the CSF and instead assuming a homogenized conductivity value inside of the skull has strong effects on EEG forward solutions, especially for superficial sources (Vorwerk et al., 2014).

These results link, elaborate upon, and validate the results of previous studies investigating the influence of conductivity uncertainties on EEG source analysis in a realistic scenario:

### 4.1. Sensitivity of EEG Forward Solutions to Conductivity Uncertainties

The results of our sensitivity analysis of the forward simulations of a P20/N20 SEP source, based on the analysis of Sobol indices, are largely in line with previous simulation-based sensitivity studies of the EEG forward problem (Gençer and Acar, 2004; Vallaghé and Clerc, 2009), finding the major sensitivity with respect to skin and skull conductivities for superficial dipole sources. Furthermore, these two studies found a notable sensitivity to the conductivity of the brain compartment (as they did not distinguish between gray and white matter). Gençer and Acar (2004) showed that this sensitivity is mainly caused by conductivity variations in the vicinity of the source. This result is in line with our findings in the five-compartment head model, which demonstrated an influence of the gray matter conductivity on the forward solution that is nearly in the range of that of the skin conductivity, whereas the influence of the white matter conductivity is noticeable only for distant electrodes. Vallaghé and Clerc (2009) also found a nonnegligible sensitivity of the EEG signal to variations of the CSF conductivity. However, these results are not in contradiction to our study but rather the consequence of using a different approach to estimate the CSF conductivity uncertainty and model variations, as they not only chose a much larger interval within which the CSF conductivity could vary (±50% compared to ±1.1% based on Baumann et al. (1997) in our study) but also had to artificially increase the thickness of the CSF compartment for computational reasons.

Besides expanding upon the results of Vallaghé and Clerc (2009) and Gençer and Acar (2004) through the additional distinction between gray and white matter, our study more importantly also represents a validation of these simulation studies using realistic data. Such validations are important to underline the relevance of the effects found in simulation studies under experimental conditions, including, for example, realistic noise patterns or inaccuracies in the model construction.

### 4.2. Influence of Conductivity Uncertainty on Dipole Reconstruction

Besides evaluations of the sensitivity of EEG forward solutions toward conductivity changes, the direct influence of (skull) conductivity changes on single dipole localizations has also been investigated in studies using both realistic (Aydin et al., 2014) and simulated data (Pohlmeier et al., 1997; Vanrumste et al., 2000; Chen et al., 2010; Gaignaire et al., 2010; Acar and Makeig, 2013). The results of these studies are in line with our findings of source localization errors in the range of centimeters due to varying skull conductivities and deeper source localizations with increasing skull conductivity. The simulation studies by Acar and Makeig (2013), Chen et al. (2010), Vanrumste et al. (2000), and Pohlmeier et al. (1997) suggest that this result is valid not only for the localization of the P20/N20 component but for focal sources throughout the cortex. Reconstructing epileptic spikes originating in the temporal lobe, Aydin et al. (2014) found location changes of more than 2 cm with varying skull conductivity. Besides a validation of the results of these previous studies, our results suggest that uncertainties of the skin conductivity may lead to noteworthy changes of the source localization, having an opposite effect to that of skull conductivity uncertainties, whereas conductivity uncertainties of gray matter, white matter, and CSF did basically not affect the source localization in our investigated scenario.

In a simulation study, Chen et al. (2010) also investigated the influence of skull conductivity changes on the reconstructed dipole orientation for source positions distributed throughout the brain and found an orientation error below 10° for the majority of sources, which is in line with our findings. However, our results (Figure 7) suggest that for the reconstruction of the P20/N20 SEP component the influence of the white matter conductivity on source orientation is equal to—if not larger than—that of the skull conductivity. Whereas we are not aware of any source reconstruction studies explicitly investigating the influence of white matter conductivity uncertainties on the reconstructed dipole orientation, a strong influence of changes of the conductivity distribution in the white matter compartment on the reconstructed source orientation for general source positions was observed in the studies of the influence of white matter anisotropy on source reconstruction results by Güllmar et al. (2010) and Hallez et al. (2008).

### 4.3. Conclusions for General Source Analysis

The results obtained in this study are directly valid only for single dipole reconstructions of a focal superficial source (P20/N20 SEP component) in the gray matter, but, in combination with the results of previous studies, it is nevertheless possible to draw conclusions for other scenarios.

#### 4.3.1. General Source Positions and Subjects

Simulation studies have shown that the effects of skull conductivity variations also hold true for general source positions (Pohlmeier et al., 1997; Vanrumste et al., 2000; Chen et al., 2010; Acar and Makeig, 2013), and Aydin et al. (2014) also found these effects specifically for sources in the temporal lobe. As the effects of skull and skin conductivity variations are linked to each other (Vallaghé and Clerc, 2009), it can be assumed that the effects of variations in skin conductivity (more superficial source localization for higher skin conductivity) also apply to general sources. However, the strength of these effects will vary for different source positions, considering that the P20/N20 component is superficial, and that the thickness of the skull changes for different brain regions. The results of Acar and Makeig (2013), analyzing the change of the dipole localization for different white matter conductivities and source positions throughout the brain, and of Wolters et al. (2006), comparing forward simulations of superficial and thalamic sources, suggest that the influence of gray matter and white matter conductivity uncertainties is stronger for deeper sources.

Furthermore, the agreement between the results in this study and those in a variety of previous studies that were performed using different head models and numerical methods suggests that the quality of the main findings, such as the opposite influence of skin and skull conductivities on the depth of the reconstructed source and the influence of the white matter conductivity on the orientation of the reconstructed source, is generalizable for arbitrary (healthy) subjects.

#### 4.3.2. Different Source Reconstruction Methods

Inverse methods for EEG can be roughly separated into two large groups: those that aim for the reconstruction of focal (dipole) sources, such as dipole fits or GFS, and those that are used to reconstruct widespread brain activity, for example to identify larger activated brain regions, such as most implementations of current density reconstruction methods (CDR). Focal sources occur, for example, for somatosensory evoked responses or in epilepsy research. In these scenarios, a highly accurate reconstruction of the source location is desired.

For the reconstruction of single, focal sources, the principal results found in this study remain valid for any inverse method that is suited for the accurate reconstruction of such dipole sources, such as dipole fits (Wolters et al., 1999; Güllmar et al., 2010) and beamformer methods (Sekihara and Nagarajan, 2008; Neugebauer et al., 2017). We further assume that the observed effects also translate to hierarchical Bayesian methods (HBM) that are suited for the localization of focal sources (Calvetti et al., 2009; Lucka et al., 2012). For multiple focal sources, we assume that the effects of conductivity uncertainties will be similar to our results in cases where the sources are clearly separated, as for example auditory evoked responses on both hemispheres, but the behavior for scenarios where the signals of multiple sources mix is unclear.

For inverse approaches that are not specifically designed for the highly accurate localization of focal dipole sources, but for the reconstruction of spatially extended sources, such as for example activated brain regions, the effects of conductivity uncertainties can be less severe than the effects observed in our study. Stenroos and Hauk (2013) showed that the source locations reconstructed using MNEs are nearly unaffected by uncertainties of the skull conductivity. Instead, variations of the skull conductivity change the amplitude and extent of the source distribution. Thus, the influence of conductivity uncertainties can possibly be ignored in many applications of MNEs. This robustness comes at the price of the “depth bias,” i.e., the reconstructed source distribution is generally located quite laterally (Lin et al., 2006), so that MNEs are not suited for the accurate spatial localization of nonsuperficial sources. For the reconstruction of deep, distributed sources, MNEs with depth-weighting have been developed that do not suffer or suffer less from depth-bias (Ioannides et al., 1990; Pascual-Marqui et al., 1994; Fuchs et al., 1999; Pascual-Marqui, 2002). Such methods are also applied in the reconstruction of relatively focal sources, e.g., in the localization of epileptic spikes (Birot et al., 2014). It is unclear how conductivity uncertainties affect the (spatial) accuracy of these methods and whether our results can be used to draw conclusions about these methods.

### 4.4. Use of SEPs for Conductivity Calibration

The results of our study confirm the choice of the skull conductivity as the focus of interest for those EEG/MEG-based conductivity estimation approaches relying on SEP/SEF measurements (Fuchs et al., 1998; Huang et al., 2007; Aydin et al., 2014; Acar et al., 2016). Based on the results of this study, the skin conductivity would be the natural candidate as an additional parameter to be fitted. However, since changes of the skin and skull conductivities showed opposing effects for many parameters (source depth, source strength, see Figures 5A, 7A), additional regularization might be necessary to achieve reliable results. The influence of gray matter and white matter conductivity uncertainties, especially on the source location, might already be too low to reliably estimate these conductivities from only P20/N20 SEP measurements.

Besides EEG/MEG-based approaches for conductivity estimation, electrical impedance tomography (EIT)-based—or aided—conductivity estimation approaches were presented, for example, by Gonçalves et al. (2003), Dabek et al. (2016), Abascal et al. (2008), Fernández-Corazza et al. (2018), and De Geeter et al. (2013). As the current for EIT is introduced at the surface of the skin, a large part of the current is channeled directly through the skin. By choosing optimal measurement patterns, the influence of the skull conductivity and of the conductivity of the brain compartments on the EIT measurement has to be maximized to achieve reliable and stable conductivity estimates. A combination of the two presented approaches, i.e., EEG/MEG- and EIT-based conductivity estimation, might lead to an additional stabilization of conductivity estimations (Pursiainen et al., 2018).

### 4.5. Limitations

The limitations in applying our results to general source reconstruction scenarios due to the use of a single dipole SEP scenario have already been discussed in detail. Although the effects and effect sizes of the influence of the respective tissue conductivity uncertainties translate to other scenarios (with certain restrictions), the specific optimal conductivity values reported in Table 3 are not. These specific values are influenced by noise in the data and the individual head model and should not be mistaken as recommendations for actual use in general source analysis scenarios.

The choice of the intervals within which each conductivity could vary, as well as the choice of the source reconstruction scenario could influence the effect sizes compared to the results observed in this study. When possible, we relied on measurement values obtained from the literature to define these intervals (skull, CSF); otherwise we determined the interval as a fixed percentage of the conductivity value, following the approach chosen in several previous studies (Haueisen et al., 1997; Vallaghé and Clerc, 2009; Schmidt et al., 2015).

With regard to possible errors in the numerical analysis, two major sources of error have to be considered: numerical errors and model simplifications. The St. Venant FEM approach was shown to lead to accurate simulation results for a correct placement of source positions within the finite element mesh (Bauer et al., 2015). We further showed that the errors caused by the use of gPC expansions are lower than the numerical errors observed for the St. Venant approach.

For the GFS with fixed and the GFS with free orientation we ensured that the dipole position was fully inside the gray matter compartment, so that a high numerical accuracy can be assumed. However, allowing sources also in the vicinity of the gray/white matter boundary for the source reconstruction with free position might lead to increased (numerical) errors. In our analysis, the majority of reconstructed source locations were inside the gray matter compartment and we did not observe any signs that the results were influenced by numerical inaccuracies, as both the results for the GoF and source depth (Figure 7) did not show any outliers. Also the localization of a few sources in the white matter did not affect our study of the influence of conductivity changes on the source localization (Figures 7, 10), as the influence of the white matter conductivity on the source localization was nevertheless found to be negligible, whereas the results with regard to the source orientation and strength were obtained for a source in the gray matter (Figures 5, 6).

Two major simplifications were applied to the head model: Ignoring the distinction between skull spongiosa and compacta and using an optimized conductivity value for the homogenized brain compartment were shown to have a negligible influence on the accuracy of the forward solutions for sources in the somatosensory cortex (Dannhauer et al., 2011; Vorwerk et al., 2014). However, this distinction should be taken into account when considering the influence of skull conductivity uncertainties for sources in the temporal lobe. Given the negligible influence of white matter conductivity uncertainties on the source localization, we assume that not modeling white matter anisotropy had a minor influence on the source localization, but it might have altered the reconstruction of the source orientation. However, whereas not modeling white matter anisotropy might lead to changes in the actually reconstructed source orientation, the variance in reconstructed source orientation should be similar for both isotropic and anisotropic modeling of the white matter. Simulation studies suggest that especially for deep sources the influence of modeling white matter anisotropy should grow (Wolters et al., 2006; Hallez et al., 2008; Güllmar et al., 2010).

Besides the model inaccuracies caused by such intentional model simplifications, unintentional model inaccuracies can also affect the source reconstruction results. For example, an accurate registration of the electrode positions to the head model is essential for accurate source localization (Dalal et al., 2014), and inaccuracies can cause significant deviations of the source reconstruction. As a consequence of model inaccuracies, the P20/N20 SEP component can be localized in the motor instead of the somatosensory cortex, corresponding to a shift by only a few millimeters, as has been previously described in the literature (Jung et al., 2008). Also, the question whether the P22 SEP component originates in the motor or in the somatosensory cortex could only recently be answered using ECoG recordings, whereas EEG source localization results were not conclusive (Baumgärtner et al., 2010).

## 5. Conclusion and Outlook

In this study, we have investigated and compared the sensitivity of EEG forward and inverse solutions for the P20/N20 SEP component to conductivity uncertainties of skin, skull, CSF, gray matter, and white matter. We found the influence of conductivity uncertainties on the source localization to be especially strong for the skull and skin compartment. However, changes of these two conductivities have opposing effects on the source localization, which has to be taken into account when performing conductivity calibrations. With regard to the reconstruction of the source orientation, the gray and white matter conductivities were found to have the strongest impact, but the observed changes of the source orientation were moderate. The variations resulting from the conductivity uncertainty for the CSF compartment do not have a recognizable impact, which is considered to be a result of the relatively small range within which the CSF conductivity varies.

In future studies, the methodology applied in this study could be used to enhance the point localizations in classical source analysis by the visualization of confidence regions or probability distributions (as done in Figure 4). The use of gPC expansions could also help to accelerate and increase the conductivity sampling density of EEG/MEG-based conductivity fitting.

## Ethics Statement

All procedures were approved by the ethics committee of the University of Erlangen, Faculty of Medicine on 10.05.2011 (Ref. No. 4453). All subjects gave written informed consent in accordance with the Declaration of Helsinki.

## Author Contributions

ÜA performed the data acquisition. JV performed the data evaluation and the computational study. All authors participated in conceiving the study, writing the paper, read and approved the final manuscript.

### Conflict of Interest Statement

CB has served as a consultant for NeuroPace, Advanced Bionics, Boston Scientific, IntelectMedical, Abbott (St. Jude Medical), and Functional Neuromodulation. CB is also a shareholder of Intelect Medical and is an inventor of several patents related to neuromodulation therapy. The remaining authors declare that the research was conducted in the absence of any commercial or financial relationships that could be construed as a potential conflict of interest.
