# Supplementary material for: Influence of Head Tissue Conductivity Uncertainties on EEG Dipole Reconstruction
Source: Front Neurosci. 2019 Jun 4;13:531. doi: 10.3389/fnins.2019.00531 (PMC6558618; doi:10.3389/fnins.2019.00531)
Supplement: Supplementary file 1 [file Data_Sheet_1.PDF]

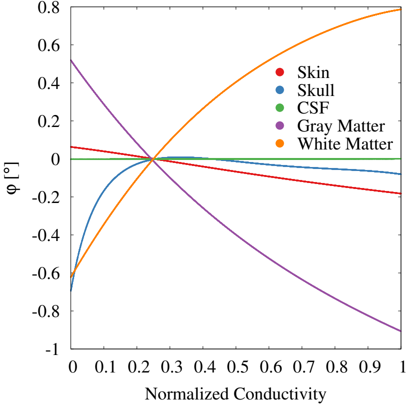

Supplementary Figure S1: Scatter plots of change in azimuthal angle (φ) for GFS with fixed dipole location and free orientation as a function of the tissue conductivity for univariate distributions. Dipole position is chosen according to the initial dipole reconstruction. Conductivities are normalized to the interval from 0 to 1 for clarity of the visualization.
